# Association Between Bladder Management Strategies and Symptomatic Urinary Tract Infections in Patients with Chronic Spinal Cord Injury: Lessons from a 5-Year Longitudinal Cohort

**DOI:** 10.3390/jcm15176626

**Published:** 2026-08-27

**Authors:** Yu-Hui Huang, Chieh-Jui Chen, Yun-Sheng Chen, Kai-Siang Chen, Sung-Lang Chen

**Affiliations:** 1 School of Medicine, Chung Shan Medical University, Taichung 402, Taiwan; yhhuang59@hotmail.com; 2 Department of Physical Medicine and Rehabilitation, Chung Shan Medical University Hospital, Taichung 402, Taiwan; 3 Department of Medical Education, Far Eastern Memorial Hospital, New Taipei 220, Taiwan; chenjerry123@gmail.com; 4 School of Medicine, College of Medicine, Taipei Medical University, Taipei 110, Taiwan; 5 Department of Obstetrics and Gynecology, Changhua Christian Hospital, Changhua 500, Taiwan; gracelucky028@gmail.com; 6 The Center of Humanities and Society, Chia Nan University of Pharmacy & Science, Tainan 717, Taiwan; cks888@mail.cnu.edu.tw; 7 Graduate Institute of Business Administration, Fu Jen Catholic University, New Taipei 242, Taiwan; 8 Department of Urology, Chung Shan Medical University Hospital, Taichung 402, Taiwan

**Keywords:** spinal cord injury, urinary tract infection, neurogenic bladder, bladder management, clean intermittent catheterization, hydronephrosis, catheter-associated urinary tract infection, cystoscopy

## Abstract

**Background:** Urinary tract infections (UTIs) are a leading cause of morbidity in patients with chronic spinal cord injury (SCI). This study examined the incidence of first and recurrent symptomatic UTIs in patients with chronic SCI and explored clinical factors associated with infection risk, with particular attention to bladder management methods and structural urinary tract abnormalities. **Methods:** We conducted a longitudinal observational study including 297 patients with chronic SCI (≥5 years post-injury; 215 males, 82 females with a mean age of 49.1 ± 13.8 years). Participants underwent regular evaluations, including kidney ultrasounds, videourodynamic studies (VUDS), and effective renal plasma flow (ERPF) measurements. Symptomatic UTIs were strictly defined using clinical–microbiological criteria to exclude asymptomatic bacteriuria (ASB). Clinical outcomes were evaluated over a minimum 5-year follow-up period. **Results:** Average annual symptomatic UTI rates were 0.31 ± 0.37 (major), 0.46 ± 0.47 (minor), and 0.77 ± 0.64 (total) episodes per patient-year. Bladder management method significantly influenced UTI rates (*p* < 0.001), with the highest rates observed in patients with indwelling urethral catheters (IUC) (1.19 ± 0.71), followed by suprapubic cystostomy (SPC) (0.91 ± 0.54), clean intermittent catheterization (CIC; 0.59 ± 0.47), and reflex voiding (0.31 ± 0.36). Recurrence (≥2 episodes) occurred in 23.9% of patients with indwelling catheters, 26.9% with suprapubic cystostomy, 13.0% with CIC, and 7.5% with reflex voiding. Patients with indwelling urethral catheters had higher total UTI rates and a higher recurrence rate than those with suprapubic cystostomy (1.19 vs. 0.91 episodes per patient-year; recurrence 38.8% vs. 26.9%). Hydronephrosis (Adjusted OR = 4.78, 95% CI: 2.45–9.32, and *p* < 0.001), bladder wall trabeculation (Adjusted OR = 3.65, 95% CI: 1.92–6.94, and *p* < 0.001), and indwelling catheter use (IUC + SPC) (Adjusted OR = 3.41, 95% CI: 1.85–6.29, and *p* < 0.001) were independently associated with increased UTI risk. **Conclusions:** Indwelling catheter use and specific structural upper and lower urinary tract abnormalities were independently associated with an increased risk of symptomatic UTIs in chronic SCI. Prioritizing CIC, routine monitoring with imaging and urodynamics, and implementing targeted preventive strategies may help reduce infection burden and preserve renal function.

## 1. Introduction

Spinal cord injury (SCI) is a major neurological disorder that may follow traumatic events such as motor vehicle accidents, falls, or violence, as well as non-traumatic etiologies including tumors, infections, and vascular disorders [1,2]. It profoundly disrupts motor, sensory, and autonomic pathways, leading to multifaceted lifelong disabilities. Among the most prevalent and burdensome secondary complications is neurogenic lower urinary tract dysfunction (NLUTD), which affects over 70–80% of individuals with SCI [3]. NLUTD manifests as impaired bladder storage, emptying, or sensation, frequently resulting in chronic urinary retention, incontinence, and elevated intravesical pressures [4]. These physiological changes contribute to urinary stasis and increase susceptibility to recurrent urinary tract infections (UTIs), which remain a leading cause of morbidity, hospitalization, and reduced quality of life in chronic SCI populations [5,6].

The annual UTI incidence in chronic SCI is reported to range from approximately 0.5 to 2.5 episodes per patient-year, with a lifetime prevalence exceeding 50–80% in many cohorts [7,8,9]. Pathophysiological mechanisms include incomplete bladder emptying (leading to urinary stasis), rapid polymicrobial biofilm formation on catheter surfaces, altered urothelial defenses, impaired immune responses, and structural sequelae such as bladder trabeculation, diverticula, vesicoureteral reflux, and hydronephrosis [7,10,11]. Recurrent infections further exacerbate the risks of antibiotic resistance, urosepsis, renal deterioration, bladder stones, and even squamous cell carcinoma of the bladder [12,13,14].

Bladder management strategies play a central role in reducing these risks. Options include clean intermittent catheterization (CIC)—preferred when feasible—indwelling urethral or suprapubic catheters, reflex (triggered) voiding, and volitional voiding in those with motor-incomplete recovery [15,16,17]. Each strategy has distinct risk–benefit profiles [8,9,10,11,12,13,14,15,16,17,18,19,20]. Adjunctive interventions, such as proactive endoscopic management of catheter debris, have shown promise in mitigating complications in patients requiring long-term indwelling catheters [21].

Despite advances in urological rehabilitation, significant gaps persist in long-term comparative data from Asian cohorts utilizing standardized symptomatic UTI definitions and serial structural evaluations [22]. While previous studies have reported higher UTI rates with indwelling catheters, this study provides additional insights through a large cohort with prospective, quarterly surveillance, strict exclusion of asymptomatic bacteriuria, multivariate adjustments for neurological and structural covariates, and integration of imaging findings. The present study aims to provide confirmatory evidence on these associations in a large cohort of patients with chronic spinal cord injury under standardized, long-term surveillance, with particular attention to both first and recurrent symptomatic infections and to structural risk factors. By addressing these associations, this study seeks informed, evidence-based, patient-centered bladder care protocols that minimize infection burden, preserve renal function, and enhance long-term outcomes in this vulnerable population.

## 2. Materials and Methods

### 2.1. Study Design

We conducted a longitudinal observational cohort study over a minimum five-year follow-up period at a tertiary specialized SCI rehabilitation and urological center in Taiwan. The study protocol adhered to the ethical standards of the Declaration of Helsinki and was approved by the Institutional Review Board of Chung Shan Medical University Hospital (CS11184) on 22 December 2011, and written informed consent was obtained from all participating patients prior to enrollment.

### 2.2. Participants

**Figure 1:**
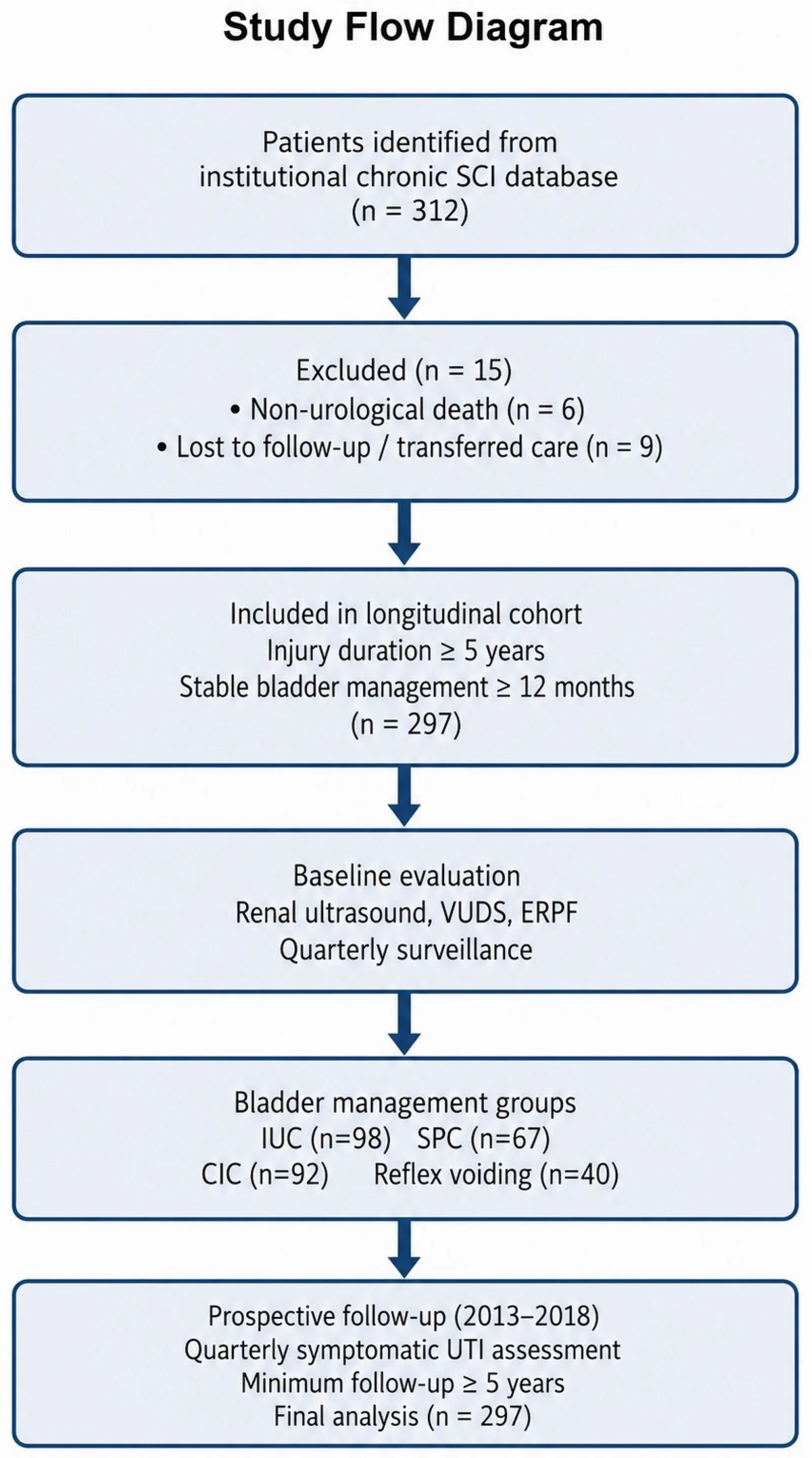
A total of 297 patients with chronic SCI were enrolled and categorized into four bladder management groups: IUC (*n* = 98), SPC (*n* = 67), CIC (*n* = 92), and reflex voiding (*n* = 40). All patients completed standardized follow-up and prospective assessment of symptomatic UTIs for at least 5 years.

Patients were identified from an existing institutional chronic SCI clinical database and then enrolled and followed prospectively with standardized quarterly assessments between 1 January 2013 and 31 December 2018 for a minimum of five years (hybrid design). Written informed consent was obtained from all participants. Initially, a total of 312 patients with chronic SCI (injury duration ≥ 5 years) were enrolled. Inclusion criteria required regular follow-up care at our institution and a stable bladder management method for at least 12 months prior to baseline. After exclusion of 6 patients who died from non-urological causes and 9 patients who were lost to follow-up or transferred care, 297 patients who completed at least five years of follow-up were included in the analysis and categorized into four bladder management groups (Figure 1). The cohort comprised 215 males and 82 females (mean age: 49.1 ± 13.8 years; mean injury duration: 15.2 ± 8.5 years). Injury levels were classified as cervical (n = 152), thoracic (n = 108), and lumbar (n = 37). In terms of completeness based on the American Spinal Injury Association (ASIA) Impairment Scale (AIS), 126 patients had complete injuries (AIS A) and 171 had incomplete injuries (AIS B–D). Patients who withdrew consent were treated as censored at the date of withdrawal, in the same manner as loss to follow-up or death. No patient withdrew consent after enrollment in the present cohort, but the handling procedure is now documented for completeness.

A substantial proportion of patients were routinely prescribed antimuscarinics or β3-agonists (mirabegron) to manage neurogenic detrusor overactivity. However, specific prescription tracking, medication adherence, and longitudinal dose adjustments were not systematically recorded in our primary database and were therefore not included as covariates in the analyses.

### 2.3. Data Collection and Surveillance Protocol

Participants underwent clinical assessments, biochemical tests, and kidney ultrasounds quarterly. Videourodynamics (VUDS) was performed every 6–12 months for high-risk patients (elevated storage pressure, previous hydronephrosis, or new symptoms) and every 12–24 months for stable low-risk patients. Effective Renal Plasma Flow (ERPF) evaluations using technetium-99m mercaptoacetyltriglycine (^99m^Tc-MAG3) renal scintigraphy were measured every 12 months for patients with known upper-tract abnormality or declining renal function, and every 24 months for stable patients.

Routine indwelling catheter exchanges (every 2 to 4 weeks according to local standard protocol) and management of catheter blockages were conducted as part of routine care. However, specific timestamps for individual catheter exchanges and acute obstruction events were not systematically logged in relation to UTI onset. UTI episodes were prospectively recorded through electronic medical records and structured patient interviews during quarterly clinic visits.

When trabeculation or hydronephrosis was detected, clinical interventions were initiated according to standard neuro-urological care, including prescribing or optimizing anticholinergic agents or $\beta_{3}$-adrenoceptor agonists (mirabegron), adjusting catheter size/drainage, and offering conversion to CIC or suprapubic cystostomy. Final management choices reflected patient agreement following shared decision-making.

#### Diagnostic Criteria for Urinary Tract Infections (UTIs)

To prevent the misclassification of asymptomatic bacteriuria (ASB)—which is highly prevalent in patients with NLUTD—symptomatic UTI was defined using strict clinical and microbiological criteria in accordance with the International Spinal Cord Injury UTI Basic Data Set and the Infectious Diseases Society of America (IDSA) guidelines.

Catheter blockage and minor UTI can present with overlapping symptoms (leakage around the catheter, suprapubic discomfort, and cloudy urine). When blockage was the clear primary event, the episode was recorded as catheter blockage and managed by catheter change and irrigation rather than being counted as a UTI. In this study, a diagnosis of minor UTI required new or acute symptoms (including, but not limited to, autonomic dysreflexia, increased muscle spasms, new onset of urinary incontinence or leaking around the catheter, suprapubic discomfort, or cloudy/foul-smelling urine with acute onset) that were not solely attributable to mechanical blockage, accompanied by both of the following:

1. Significant pyuria, which is defined as ≥10 white blood cells (WBCs) per high-power field (HPF) on urine microscopy or a positive leukocyte esterase test on urinalysis.

2. A positive urine culture demonstrating significant bacteriuria (≥10^3^ colony-forming units [CFU]/mL for patients performing CIC and ≥10^2^ CFU/mL for patients with indwelling urethral or suprapubic catheters).

The diagnosis of a major UTI required the presence of systemic signs or symptoms, including documented fever (>38.0 °C), rigors, systemic inflammatory response syndrome (SIRS), or clinical deterioration requiring hospitalization, together with a microbiologically confirmed positive urine culture. Asymptomatic bacteriuria, characterized by positive urine cultures in the absence of acute clinical signs or symptoms, was systematically excluded from the UTI incidence calculations.

### 2.4. Statistical Analysis

Descriptive statistics summarized clinical and demographic characteristics. UTI rates were expressed as episodes per patient-year. Continuous variables were compared using one-way ANOVA followed by Bonferroni post hoc tests for pairwise comparisons. Categorical variables and rates were evaluated using chi-square tests. Point-biserial correlation coefficients (r) were used to evaluate linear associations between continuous clinical markers.

Because UTI rates (episodes per patient-year) are count data, we assessed overdispersion before model selection. The dispersion statistic indicated overdispersion; therefore, negative binomial regression with Bonferroni-adjusted pairwise contrasts was used as the primary method to compare rates across bladder management groups. One-way ANOVA results are presented only as a sensitivity analysis.

A multivariate logistic regression model was utilized to assess independent predictors of frequent symptomatic UTIs (≥1 episode/year). Candidate covariates were chosen based on both clinical relevance and the results of univariate analysis (*p* < 0.20) and the prior literature; these included age, sex, neurological level, AIS Impairment Scale grade, injury duration, bladder management strategy, hydronephrosis, bladder wall trabeculation, and bladder compliance. The final model demonstrated acceptable discrimination (c-statistic = 0.78, 95% CI 0.72–0.84) and good calibration (Hosmer–Lemeshow test, *p* = 0.312). Multicollinearity was absent (all VIFs < 2.0).

The sample size of 297 was determined by consecutive enrollment of all eligible patients who completed at least five years of follow-up. An a priori sample-size calculation was not performed. In the primary multivariable logistic regression model for frequent UTI (≥1 episode/year), the number of outcome events was sufficient to yield an events-per-variable (EPV) ratio exceeding the conventional threshold of 10. The relatively wide confidence interval in the smallest subgroup (reflex voiding, n = 40) is noted as a limitation of precision for that comparison.

We used Kaplan–Meier survival analysis to evaluate the time to the first symptomatic UTI, with the time to the second event assessed in a secondary analysis, stratified by baseline bladder management modality, and compared using the log-rank test. Bladder management was analyzed as a time-fixed baseline variable because patients were required to have a stable method for at least 12 months prior to enrollment. Temporary short-term deviations (for example, during an acute illness) were not regarded as a change in primary strategy. Patients who permanently switched their long-term method during follow-up were either excluded from the analytic cohort or censored at the time of the switch, thereby preserving the integrity of the four baseline groups. All statistical analyses were conducted using SPSS software (v26.0; IBM Corp., Armonk, NY, USA), with significance set at *p* < 0.05.

## 3. Results

### 3.1. Participant Demographics

A total of 297 patients with chronic SCI (injury duration ≥5 years) were included in the final analysis. The cohort consisted of 215 males (72.4%) and 82 females (27.6%), with a mean age of 49.1 ± 13.8 years and a mean injury duration of 15.2 ± 8.5 years. Injury levels were classified as cervical in 152 patients (51.2%), thoracic in 108 patients (36.4%), and lumbar in 37 patients (12.5%). Based on the American Spinal Injury Association (ASIA) Impairment Scale, 126 patients (42.4%) presented with complete injuries (AIS A), while 171 patients (57.6%) had incomplete injuries (AIS B–D). Baseline demographic and injury characteristics are summarized in Table 1.

### 3.2. UTI Incidence Rates

Over the five-year follow-up period, a total of 1143 symptomatic UTI episodes were confirmed. The overall cohort average annual UTI rate was 0.77 ± 0.64 episodes per patient-year. When stratified by severity, minor UTIs predominated (0.46 ± 0.47 episodes/year), while major UTIs requiring hospitalization or presenting with systemic fever occurred at a lower rate (0.31 ± 0.37 episodes/year).

### 3.3. Bladder Management and UTI Frequency

Bladder management method was significantly associated with overall annual symptomatic UTI rates (negative binomial regression, overall *p* < 0.001). One-way ANOVA yielded the same overall conclusion and is presented as a sensitivity analysis. Patients managed with indwelling urethral catheters demonstrated the highest infection rates (1.19 ± 0.71 episodes per patient-year), followed by those with suprapubic cystostomy (0.91 ± 0.54), CIC (0.59 ± 0.47), and reflex voiding (0.31 ± 0.36). Direct comparison showed higher total UTI rates and higher recurrence in the urethral-catheter group than in the suprapubic-cystostomy group. Pairwise comparisons using Bonferroni post hoc adjustment confirmed that rates in the indwelling urethral catheter, suprapubic cystostomy, and reflex voiding groups showed statistically significant differences from those in the CIC group (Table 2).

### 3.4. Structural Risk Factors and Multivariate Analysis

Our radiological and urodynamic reviews identified substantial variations in upper and lower urinary tract structural integrity. Bladder wall trabeculation was observed in 143 patients (48.1%), while hydronephrosis was documented in 61 patients (20.5%).

Point-biserial correlation analysis showed strong positive associations between high annual UTI frequency and both hydronephrosis (r = 0.74, *p* < 0.001) and bladder wall trabeculation (r = 0.67, *p* < 0.001). Poor bladder compliance (defined as <15 mL/cmH_2_O) showed a weak, non-significant linear correlation (r = 0.11, *p* = 0.09).

In the multivariate logistic regression analysis modeling the risk of experiencing frequent symptomatic UTIs (≥1 episode/year), hydronephrosis, bladder wall trabeculation, and indwelling catheter use remained robust independent predictors (Table 3).

Dichotomization of the outcome (≥1 episode/year) discards information on the actual number of events, within-subject correlation, and variable follow-up duration.

The model demonstrated good calibration, with the Hosmer–Lemeshow test yielding a *p*-value greater than 0.05, and no evidence of clinically significant multicollinearity was observed among the included covariates (all VIFs < 2.0). Variables including age, sex, neurological level, AIS grade, and injury duration were not independently associated with frequent symptomatic UTIs after adjustment.

### 3.5. Kaplan–Meier Survival Analysis for UTI-Free Survival

**Figure 2:**
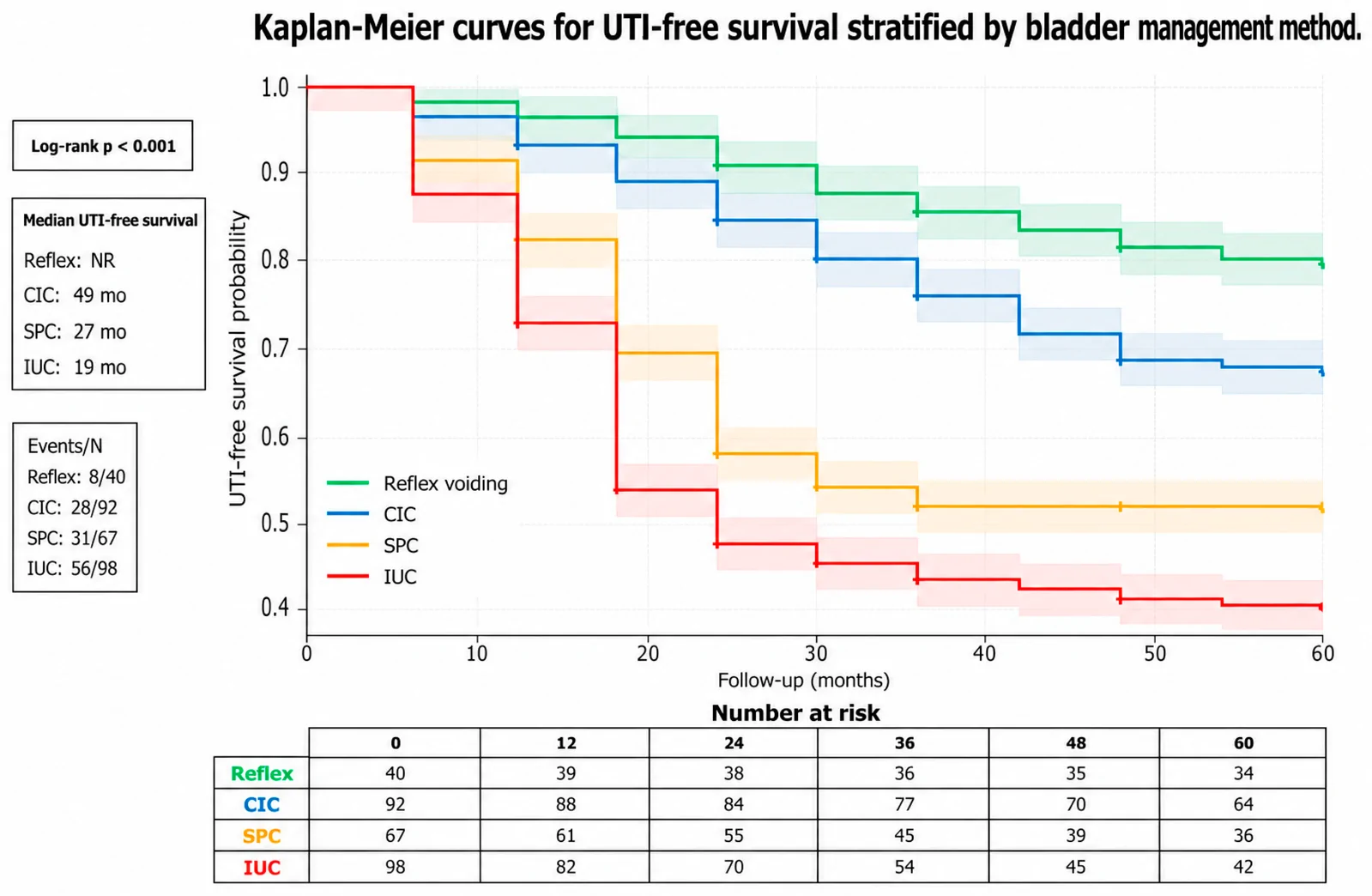
Kaplan–Meier curves for UTI-free survival stratified by bladder management method. Patients managed with reflex voiding and CIC demonstrated significantly better UTI-free survival than those managed with SPC or IUC. Shaded regions indicate 95% confidence intervals, and censoring is indicated by plus signs (+). Median UTI-free survival was 19 months for IUC, 27 months for SPC, and 49 months for CIC, whereas the median survival was not reached for reflex voiding. Log-rank test: *p* < 0.001. CIC, clean intermittent catheterization; SPC, suprapubic cystostomy; IUC, indwelling urethral catheter; UTI, urinary tract infection.

Kaplan–Meier analysis of time to first symptomatic UTI episode over the 5-year study period showed statistically significant differences among the bladder management groups (log-rank test *p* < 0.001) (Table 4 and Figure 2).

### 3.6. UTI Recurrence Analysis

Given that recurrent UTIs are of greater clinical concern than isolated events in the chronic SCI population, we further analyzed recurrence patterns. Overall, 123 patients (41.4%) experienced at least one symptomatic UTI, and 71 patients (23.9%) experienced recurrent UTIs (≥2 episodes during follow-up).

**Figure 3:**
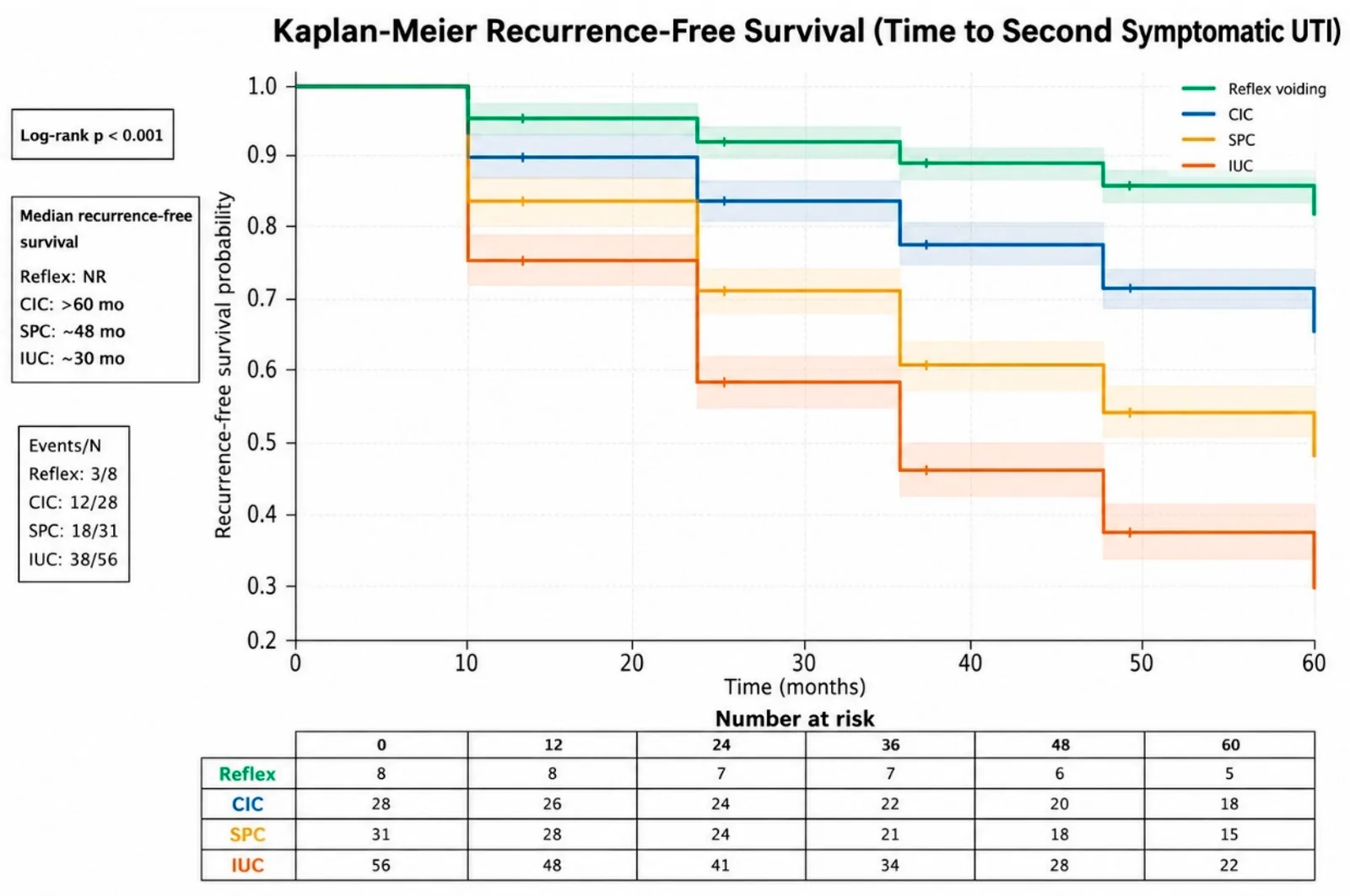
Kaplan–Meier analysis of recurrence-free survival (time to second symptomatic UTI) according to bladder management method. Recurrence-free survival differed significantly among groups (log-rank *p* < 0.001). In-dwelling urethral catheterization was associated with the shortest recurrence-free survival, whereas reflex voiding demonstrated the longest recurrence-free survival. Shaded bands indicate 95% confidence intervals and plus signs (+) denote censored observations. Numbers at risk are shown below the x-axis. CIC, clean intermittent catheterization; SPC, suprapubic cystostomy; IUC, indwelling urethral catheter; UTI, urinary tract infection.

Recurrence rates differed among bladder management methods (χ^2^ test, *p* < 0.001). Patients with indwelling urethral catheters had the highest proportion of recurrent UTIs (38.8%, 95% CI 29.5–48.9%), followed by suprapubic cystostomy (26.9%, 95% CI 17.3–39%), CIC (13.0%, 95% CI 7.5–21.5%), and reflex voiding (7.5%, 95% CI 2.6–19.9%) (Table 5 and Figure 3).

A multivariable logistic regression model for recurrent UTIs (≥2 episodes) was used with the same covariate set as the model in Table 3. In addition to hydronephrosis, bladder wall trabeculation and indwelling catheter use also remained independently associated with higher odds of recurrence (Adjusted OR 3.87, 95% CI 2.12–7.05, and *p* < 0.001). These findings confirm that bladder management modality strongly influences not only first-event rates but also the burden of recurrent infection.

### 3.7. Urodynamic and Renal Function Parameters by Bladder Management Group

For the analyses in Table 6, patients managed with an indwelling urethral catheter (n = 98) and those managed with a suprapubic cystostomy (n = 67) were combined into a single “Indwelling Catheter” group (n = 165) and compared with patients managed by clean intermittent catheterization or reflex voiding (n = 132).

Key urodynamic and renal function parameters differed substantially according to bladder management method (Table 6). Patients managed with indwelling catheters (urethral or suprapubic) exhibited higher detrusor leak point pressures (57.6 ± 19.4 vs. 29.4 ± 14.2 cmH_2_O), lower maximum cystometric capacity (248 ± 132 vs. 392 ± 141 mL), lower effective renal plasma flow (274 ± 85 vs. 372 ± 78 mL/min/1.73 m^2^), and a markedly higher prevalence of hydronephrosis (32.7% vs. 5.3%) compared with those managed by CIC or reflex voiding (all *p* < 0.001).

### 3.8. Microbiological Profile of Major UTIs

A total of 312 culture-positive major UTI episodes were analyzed. The most frequently isolated pathogens are summarized in Table 7. *Escherichia coli* was the predominant organism (41.7%), followed by *Klebsiella pneumoniae* (19.2%) and *Pseudomonas aeruginosa* (13.5%). Extended-spectrum beta-lactamase (ESBL) production was common among Enterobacterales, and carbapenem resistance was observed particularly in *Pseudomonas aeruginosa* (12.4%).

## 4. Discussion

In this cohort of 297 patients with chronic SCI, the hierarchy of UTI risk according to bladder management method (indwelling urethral catheter > suprapubic cystostomy > clean intermittent catheterization > reflex voiding) is consistent with earlier reports. Our findings therefore serve primarily as confirmatory evidence from a large, well-characterized Asian population followed with rigorous exclusion of asymptomatic bacteriuria and regular imaging and urodynamic assessment. A relatively distinctive observation in the present study is that the differences among bladder management strategies were also more pronounced for recurrent UTIs than for first events (Table 5 and Figure 3). This suggests that long-term management strategies may have a cumulative impact on the burden of repeated infections as on the occurrence of a single first episode.

### 4.1. Comparison of UTI Rates Across Bladder Management Modalities

Bladder management strategy was strongly associated with both first event and recurrent symptomatic UTI rates in this cohort (*p* < 0.001). Indwelling urethral catheters were associated with the highest infection burden (1.19 ± 0.71 episodes per patient-year), consistent with previous reports linking chronic transurethral catheterization to elevated UTI risk, largely attributable to rapid biofilm formation [23,24]. Suprapubic cystostomy showed a modest advantage over urethral catheters (0.91 vs. 1.19 episodes/year), in line with earlier Taiwanese data [25], primarily by reducing urethral trauma [26,27]. CIC was associated with substantially lower rates (0.59 ± 0.47 episodes/year), supporting current EAU and CUA guidelines that recommend CIC as the preferred strategy whenever clinically feasible [3,28].

The same risk hierarchy (indwelling urethral catheter > suprapubic cystostomy > CIC > reflex voiding) was observed for both first symptomatic UTIs and recurrent infections, but the differences were more pronounced for recurrence. First-event rates ranged from 1.19 to 0.31 episodes per patient-year. In contrast, the proportion of patients experiencing recurrent UTIs (≥2 episodes) was 38.8% with indwelling catheters, 26.9% with SPC, 13% with CIC, and only 7.5% with reflex voiding (Table 5). Median time to the second UTI was correspondingly shorter in the indwelling group (30 months) than in the reflex-voiding group (>60 months; log-rank *p* < 0.001; Figure 3). The differences among bladder management methods were also pronounced for recurrent infections as for first events, suggesting that long-term management strategy is particularly important in patients with repeated UTIs.

### 4.2. Structural Sequelae and Upper Tract Deterioration

Beyond differences in bladder management, this study established strong positive correlations between high UTI frequency and advanced structural remodeling—namely hydronephrosis (r = 0.74) and bladder wall trabeculation (r = 0.67). In chronic NLUTD, sustained high storage pressures and detrusor-sphincter dyssynergia promote gradual hypertrophy of the detrusor smooth muscle, clinically and morphologically evidenced by bladder trabeculation [3,8,29].

Aligning with prior research, our results indicate that hydronephrosis and structural bladder remodeling correlate with recurrent urinary tract infections and upper urinary tract deterioration in patients with SCI [18,30,31]. Urinary stasis within diverticula and trabeculated areas may facilitate bacterial persistence and recurrent infection. Structural abnormalities may contribute to urinary stasis, bacterial persistence, and recurrent infection, which in turn may further impair upper urinary tract function [31]. Current EAU and AUA/SUFU guidelines recommend periodic upper urinary tract imaging and VUDS assessment in patients with chronic NLUTD, and our results are in line with these recommendations [3,8,28,32]. Patients with indwelling catheters had markedly higher detrusor leak point pressures, reduced bladder capacity, and lower ERPF values (Table 6), consistent with greater risk of upper tract deterioration. These urodynamic and renal function findings were consistent with the observed association between structural abnormalities and recurrent symptomatic UTIs.

### 4.3. Clinical Implications, Risk Factors and Prevention Strategies

The results suggest that bladder management should be individualized according to neurological status, bladder function, and patient preference. CIC should remain the preferred strategy whenever feasible, whereas patients requiring chronic indwelling catheterization may benefit from closer surveillance with renal ultrasonography and VUDS [3,8,28,32]. Detection of hydronephrosis or bladder trabeculation may identify patients at particularly high risk for recurrent symptomatic UTIs [16,18,30]. In selected patients with chronic indwelling catheters and significant intravesical debris, adjunctive cystoscopic removal may also be considered as part of a comprehensive management strategy [21]. Removal of crystalline deposits and mucoid debris may reduce catheter obstruction and bacterial persistence, thereby lowering the risk of symptomatic infection. This strategy may represent a useful adjunctive approach for high-risk patients with indwelling catheters who suffer from recurrent blockages. These findings support further evaluation of this strategy in randomized studies. The microbiological findings (Table 7) showed that Gram-negative organisms predominated, with a substantial proportion exhibiting ESBL production or carbapenem resistance, underscoring the need for antimicrobial stewardship alongside mechanical interventions such as proactive cystoscopic debris removal [6,10,24].

Recent guidelines have placed greater emphasis on shared decision-making, telemedicine, and emerging technologies in long-term SCI care [3,8,24,28]. Strict focus on symptomatic UTIs (distinguishing from asymptomatic bacteriuria) is essential.

### 4.4. Methodological Considerations and Primary Model Limitations

Using standard binary logistic regression and single-event survival models in our primary analysis collapses detailed longitudinal records into fixed, binary endpoints (e.g., presence or absence of ≥1 UTI episode per year). While this yields clear odds ratios and time-to-first-event estimates, it discards key clinical data: it ignores total event counts, omits within-patient event clustering over time, and treats variable follow-up durations as uniform intervals. Because advanced count models (such as negative binomial regression) and recurrent-event survival models (such as Andersen–Gill or frailty Cox proportional hazards) were not applied as the primary framework across all secondary outcomes, our reported effect estimates reflect threshold risk rather than total cumulative disease burden. Readers should interpret the absolute magnitude of these effect sizes with caution, recognizing that multi-state and repeated-measures frameworks remain the statistical standard for capturing the true, dynamic trajectory of recurrent UTIs in chronic SCI cohorts.

### 4.5. Interpretation of the Lower Symptomatic UTI Incidence Compared with Previous Studies

The overall symptomatic UTI incidence in our cohort (0.77 episodes per patient-year) was lower than the approximately 0.5–2.5 episodes per patient-year reported in several earlier SCI cohorts [7,9,23,33]. This difference is likely explained, at least in part, by variations in study design and UTI definitions. Our study adopted stringent diagnostic criteria requiring compatible clinical symptoms, pyuria, and microbiological confirmation while systematically excluding asymptomatic bacteriuria [3,6]. Furthermore, all participants received structured follow-up within a specialized neuro-urological program, including regular imaging and VUDS surveillance, which may have contributed to earlier identification of urinary tract abnormalities and optimized bladder management [16,30]. Therefore, direct comparison of incidence rates across studies should be interpreted cautiously because reported UTI rates are highly influenced by diagnostic criteria, surveillance intensity, bladder management practices, and patient characteristics. Future multicenter studies using standardized UTI definitions are needed to improve comparisons across SCI populations [3,6,8].

### 4.6. Take-Home Messages for Clinical Practice and Patient Care

Real-world surveillance reveals a substantial baseline prevalence of hydronephrosis (20.5%) and bladder-wall trabeculation (48.1%) in chronic SCI populations, underscoring the necessity of routine upper-tract imaging regardless of reported symptoms. Bladder management reflects patient-centered choices that balance clinical risk against practical factors such as hand dexterity, caregiver support, personal lifestyle and local infrastructure; when patients cannot or choose not to transition to CIC, proactive surveillance and aggressive medical management of storage pressures become critical. Indwelling catheterization carries a significantly higher risk of recurrent urinary tract infections compared with intermittent catheterization and reflex voiding, highlighting the need to minimize reliance on indwelling catheters whenever clinically feasible. For patients who must remain on indwelling catheters, proactive attention to catheter hygiene, minimization of exchange-related trauma, and prevention of encrustation or blockage through regular flushing are crucial to lowering sudden acute infection spikes.

### 4.7. Study Limitations

While our study’s key attributes include a sizeable patient cohort (n=297) and an extended longitudinal follow-up of at least five years, several notable limitations warrant acknowledgment.

First, as a single-center study conducted within a specialized urological rehabilitation facility in Taiwan, our findings may reflect localized patient-care protocols, intensive surveillance (quarterly renal ultrasound and periodic VUDS), and referral patterns. These factors may limit the generalizability of our findings to healthcare settings with different follow-up practices. Second, the observational nature of this cohort introduces substantial confounding by indication. Patients managed with indwelling urethral or suprapubic catheters were fundamentally different from those using CIC or reflex voiding—typically presenting with higher rates of complete cervical injuries, poorer upper-extremity function, greater neurological impairment, and more severe bladder dysfunction. Although multivariable logistic regression was adjusted for neurological level, AIS grade, age, sex, injury duration, and structural variables, residual confounding cannot be excluded. Propensity-score matching or inverse-probability weighting was not performed. Accordingly, the observed differences should be interpreted as associations rather than evidence of causality. Third, the primary analytic approach dichotomized the outcome into frequent UTIs (≥1 episode/year). This binary logistic regression discards information on the number of recurrent events, within-subject correlation, and varying follow-up times. More appropriate recurrent-event models (for example, negative binomial regression, Andersen–Gill, or frailty Cox models) were not applied and represent an important direction for future analyses. Fourth, bladder management was treated as a time-fixed baseline variable because patients were required to maintain a stable method for at least 12 months prior to enrollment. Changes in management during follow-up, death as a competing risk, and potential immortal-time bias were not fully modeled in the Kaplan–Meier analyses. Fifth, while we strictly adhered to clinical–microbiological criteria to distinguish symptomatic UTIs from asymptomatic bacteriuria, comprehensive longitudinal data on uropathogen distribution, multidrug resistance, and antibiotic prescribing patterns were not systematically analyzed. A minority of patients received intermittent or continuous low-dose antibiotic prophylaxis during the five-year observation period, usually prescribed by their community physicians for recurrent symptomatic infections. Because prophylaxis was neither protocolized nor systematically recorded as a study variable, it was not included as a covariate in the main analyses and could not be evaluated as a potential confounder. Finally, procedural factors related to indwelling catheters—such as post-exchange UTIs resulting from mucosal trauma or infections secondary to acute catheter blockages—were not individually tracked. Catheter encrustation and exchange-related bacterial inoculation remain important clinical determinants of catheter-associated urinary tract infection (CAUTI) that warrant granular tracking in future prospective trials.

## 5. Conclusions

Our findings support individualized bladder management strategies that prioritize CIC whenever clinically feasible in patients with chronic SCI. Patients requiring chronic indwelling catheterization may benefit from routine imaging surveillance and VUDS evaluation to identify structural abnormalities associated with an increased risk of symptomatic UTIs. This study found that bladder management method and structural urinary tract abnormalities were independently associated with first and recurrent symptomatic UTI risk in chronic SCI. Additional multicenter studies using recurrent-event models and methods to reduce confounding are needed to confirm these findings.

## Figures and Tables

**Table 1:** Baseline demographic and clinical characteristics of the study population (n = 297).

Characteristic	Value	Percentage (%)
Sex		
Male	215	72.4
Female	82	27.6
Neurological level of injury		
Cervical	152	51.2
Thoracic	108	36.4
Lumbar	37	12.5
AIS grade		
Complete injury (AIS A)	126	42.4
Incomplete injury (AIS B–D)	171	57.6
Age, years	49.1 ± 13.8	—
Injury duration, years	15.2 ± 8.5	—

Data are presented as numbers (percentages) or mean ± standard deviation. AIS = American Spinal Injury Association Impairment Scale.

**Table 2:** UTI rates by bladder management method.

Bladder Management Method	n	Total UTI Rate (Mean ± SD)	Major UTI Rate	Minor UTI Rate	Rate Ratio vs. CIC (95% CI)	*p*-Value
Indwelling Urethral Catheter	98	1.19 ± 0.71	0.47	0.72	2.02 (1.56–2.61)	<0.001
Suprapubic Cystostomy	67	0.91 ± 0.54	0.34	0.57	1.54 (1.15–2.07)	0.004
Clean Intermittent Catheterization	92	0.59 ± 0.47	0.21	0.38	Reference	—
Reflex Voiding	40	0.31 ± 0.36	0.11	0.20	0.53 (0.35–0.80)	0.003

UTI rates are expressed as episodes per patient-year (mean ± SD). Rate ratios and *p*-values from negative binomial regression with Bonferroni-adjusted pairwise comparisons versus the CIC group. UTI rates are expressed as episodes per patient-year.

**Table 3:** Multivariate logistic regression for factors associated with frequent UTIs (≥1 episode/year).

Risk Factor	Point-Biserial r	Adjusted Odds Ratio (95% CI)	*p*-Value
Hydronephrosis	0.74	4.78 (2.45–9.32)	<0.001
Bladder Wall Trabeculation	0.67	3.65 (1.92–6.94)	<0.001
Indwelling Catheter Use (vs. Non-Indwelling)	—	3.41 (1.85–6.29)	<0.001
Poor Bladder Compliance	0.11	1.21 (0.68–2.15)	0.521

Adjusted odds ratios (OR) with 95% confidence intervals (CI) are shown. The model included age, sex, neurological level, AIS grade, injury duration, bladder management method, hydronephrosis, bladder wall trabeculation, and bladder compliance. Point-biserial correlation coefficients (r)P with annual UTI frequency are also presented for continuous variables.

**Table 4:** Summary of 5-year UTI-free survival stratified by bladder management.

Bladder Management Group	Median UTI-Free Survival (Months)	5-Year UTI-Free Survival Rate (%)	Log-Rank *p*-Value
Indwelling Urethral Catheter	19 months	43.0%	<0.001 (overall)
Suprapubic Cystostomy	27 months	54.0%	—
Clean Intermittent Catheterization	49 months	70.0%	—
Reflex Voiding	>60 months	81.0%	—

Kaplan–Meier analysis was used to estimate median urinary tract infection (UTI)-free and 5-year survival rates, with overall comparisons conducted via the log-rank test.

**Table 5:** UTI recurrence rates by bladder management method.

Bladder Management Method	n	≥1 UTI n (%)	Recurrent (≥2) n (%)	95% CI for Recurrence	Mean Episodes Among Recurrent (Mean ± SD)
Indwelling Urethral Catheter	98	56 (57.1)	38 (38.8)	29.5–48.9	3.9 ± 1.8
Suprapubic Cystostomy	67	31 (46.3)	18 (26.9)	17.3–39.0	3.4 ± 1.6
Clean Intermittent Catheterization	92	28 (30.4)	12 (13.0)	7.5–21.5	2.7 ± 1.3
Reflex Voiding	40	8 (20.0)	3 (7.5)	2.6–19.9	2.0 ± 0.0
Overall	297	123 (41.4)	71 (23.9)	19.4–29.1	3.5 ± 1.7

Recurrent UTI was defined as ≥2 symptomatic episodes during the follow-up period. The number of patients with ≥1 UTI matches the first-event counts from the Kaplan–Meier analysis (Figure 2 and Table 4). 95% confidence intervals for recurrence proportions were calculated using the Wilson method. Overall comparison of recurrence rates across groups was performed using the χ^2^ test (*p* < 0.001).

**Table 6:** Key urodynamic and renal function parameters by bladder management group.

Parameter	Overall (n = 297)	Indwelling Catheter (n = 165)	CIC/Reflex (n = 132)	*p*-Value
Detrusor Leak Point Pressure (cmH_2_O, mean ± SD)	44.8 ± 21.3	57.6 ± 19.4	29.4 ± 14.2	<0.001
Maximum Cystometric Capacity (mL)	312 ± 148	248 ± 132	392 ± 141	<0.001
Effective Renal Plasma Flow (ERPF, mL/min/1.73 m^2^)	318 ± 92	274 ± 85	372 ± 78	<0.001
Hydronephrosis (any grade)	20.5%	32.7%	5.3%	<0.001

Data are presented as mean ± standard deviation or percentage. Indwelling catheter group includes both urethral and suprapubic catheters. Comparisons were performed using independent *t*-tests or chi-square tests as appropriate. ERPF = effective renal plasma flow.

**Table 7:** Most common pathogens isolated in major UTIs (n = 312 culture-positive episodes).

Pathogen	Frequency (%)	ESBL-Producing (%)	Carbapenem-Resistant (%)
*Escherichia coli*	41.7	26.9	4.6
*Klebsiella pneumoniae*	19.2	31.7	7.8
*Pseudomonas aeruginosa*	13.5	–	12.4
*Enterococcus* spp.	9.6	–	–
*Proteus mirabilis*	7.1	18.2	–
Other (including MDR organisms)	8.9	22.1	9.3

Percentages refer to the proportion of culture-positive major UTI episodes. ESBL = extended-spectrum β-lactamase; MDR = multidrug-resistant.

## Data Availability

The data presented in this study are available on request from the corresponding author with a reasonable reason.
